# Effectiveness of the graded transport mode for the intrahospital transport of critically ill patients: A retrospective study

**DOI:** 10.3389/fpubh.2022.979238

**Published:** 2023-01-13

**Authors:** Lijing Ling, Xiaohua Xia, Hua Yuan, Shifang Liu, Zhiqiang Guo, Caihong Zhang, Jin Ma

**Affiliations:** Department of Emergency Medicine, Affiliated Kunshan Hospital of Jiangsu University, Kunshan, China

**Keywords:** emergency treatment, critically ill patients, intrahospital transport, adverse events, graded transport

## Abstract

**Aim:**

The purpose of this study was to evaluate the effectiveness of the graded transport mode in the intrahospital transport (IHT) of critically ill patients.

**Methods:**

This is a retrospective study, including 800 patients and categorized them into control and observation groups. The control group included 420 critically ill patients who were transported *via* conventional methods from our emergency resuscitation unit from June 2017 to December 2017. The observation group included 380 critically ill patients who were transported through a graded transport mode from January 2018 to June 2018. We performed intergroup comparisons of the incidence rates and causes of adverse events (AEs), transport time, length of stay, and mortality rate.

**Results:**

The observation group had significantly lower transport time and AE incidence rates than the control group. However, no significant differences were observed in terms of the length of stay and mortality rate between the two groups.

**Conclusion:**

The most notable merits of the graded transport mode in the IHT of critical care patients include the fact that it significantly reduces the incidence of AEs during IHT, shortens the transport time, and improves transport efficiency, thereby ensuring the safety of critically ill patients.

## Introduction

The emergency resuscitation room is the first admission unit for critically ill patients; patients whose condition does not improve after the initial evaluation and resuscitation need to be transported to appropriate departments for further diagnosis, examination, treatment, and hospitalization, known as intrahospital transport (IHT) (1). IHT exposes patients to a mobile environment and increases their susceptibility to the occurrence of adverse events (AEs). Severe AEs can exacerbate diseases and even endanger patient lives and are associated with significant mortality and increased healthcare costs. To ensure the timeliness and safety of IHT for these patients, standardization and optimization of the IHT process are urgently required (2, 3).

In recent years, graded transport mode, as a new IHT method, has been widely applied in Chinese hospitals. According to the severity of patients, the graded transport mode divides IHT into three risk levels, namely Grade I (high risk), Grade II (medium risk), and Grade III (low risk). However, the differences in transport time, length of hospital stay, and mortality between graded transport mode and traditional transport mode remain unknown.

In this study, we developed a new set of quality standards for IHT in critically ill patients. We aimed to explore whether the graded transport mode can effectively reduce the incidence of IHT related adverse events, shorten transport time and improve transport efficiency.

## Methods

### Sample sources

We collected critically ill patients admitted to the emergency department of our hospital from June 2017 to June 2018 and divided them into graded transport and traditional transport. We selected patients according to the following inclusion criteria: (1) critically ill patients admitted to the emergency resuscitation room requiring IHT owing to the need for CT and other imaging examinations, emergency interventions, emergency surgery, and hospitalization; (2) age > 18 years; (3) signed informed consent for the risk of transport obtained from the families of all patients; and (4) complete medical records. Exclusion criteria were pregnancy, infectious disease, hospital transfer, and patients with missing values.

### IHT modes

Patients in the control group received conventional transport. After the attending physician assessed the patients' condition and permitted the transport, the families of the patients were involved in the transport process. In addition, emergency nurses and physicians carried respiratory support, resuscitation supplies, cardiac monitors, and other facilities for assistance.

Patients in the observation group received the graded transport. The steps of graded transport are as follows: (1) evaluation of transport risk grading: the transport risk grading is determined according to the patient's condition classification standard, and the corresponding medical staff, instruments, equipment and drugs are allocated according to different risk levels. Classification criteria are mainly referred to Vital Signs, Glasgow Coma Scale (GCS) score for consciousness state, Respiratory support, Circular support and primary clinical symptoms; (2) preparation before transport: the nurse in charge of transport must check the patient's physical status, equipment, drugs, etc., and fill in the form for graded transport; (3) transport: during the transport, the patient's physical condition and condition change must be observed at all times, ensure the safe placement and normal operation of medical equipment, prepare for emergency treatment, and try to avoid the occurrence of adverse events in transport; and (4) end of transport: the nurse in charge of transfer shall evaluate the situation of the patient after transfer and record the relevant contents of graded transport. Finally, the doctor in charge of the transfer and the doctor of the department receiving the patient jointly confirm the completion of the whole transfer process. Transport risk was categorized into Grades I, II, and III (from high to low), and the specific measures are presented in Tables 1, 2.

**Table 1:** Specific measures related to intrahospital transport for critically ill patients in the control and observation groups.

**Measures**	**Control group (June 2017 to December 2017)**	**Observation group (January 2018 to June 2018)**
Transport training	(1) Maintenance of transport-related apparatus and equipment; (2) Ensuring the presence of an emergency plan for cardiac arrest, accidental extubation of artificial airway, malfunction of the ventilator during transport, and insufficient oxygen source; (3) Three theoretical lectures on cardiopulmonary resuscitation (CPR) operation during transfer and team first aid; (4) Nurses and physicians are required to have worked in emergency medicine for more than 1 year and 6 months, respectively. In addition, they are required to attend training and qualify in the theoretical, CPR, and IHT simulation tests in emergency medicine before they can be deemed independent in performing IHT for critically ill patients	(1) In addition to the traditional training methods, ensuring the inclusion of special safety inspection training modules including the DSA safety instruction, enhanced CT safety instruction, and hyperbaric oxygen safety instructions; (2) Emphasis on training on the use of transport vehicles, apparatus, and drugs (all participants need to qualify in the examination); (3) Emphasis on the transport mode in the status, background, assessment, and recommendations
Risk grading	Comprehensive assessment of vital signs by the attending physician	Grades I, II, and III (Table 2)
Logistical support	Dedicated transport elevators equipped with a dedicated operating staff	(1) Relatively fixed transport logistics staff are trained; (2) The number of transport elevators is increased each building is installed with a dedicated elevator for transport; ensuring that elevator staff members wear identification badges, carry wireless intercom to contact in advance to prepare for transport, and possess excellent service attitudes; (3) Emergency drills for safe transport are regularly performed
Communication and cooperation	Routine telephone communication between medical and nursing staff and relevant departments	(1) Efficient communication between the physician and the examining department to reduce waiting time. (2) Health education and psychological care by nurses to calm anxious family members of patients; (3) Transport operation by experienced medical personnel; (4) Radiological examination report should be issued within 15 min (examination report for special patients should be issued immediately); (5) Verification of patient, drug, and catheter information by medical personnel when handing over patients to other departments
Quality control analysis	Quarterly quality control analysis of the pertinent problems, adjustments, and improvements	(1) Lectures are conducted to train medical staff to identify the disease etiologies; (2) Transport defect analysis for critically ill patients is performed once a month (defects were analyzed using a simple root cause analysis method to reduce risk)

**Table 2:** Transport risk grading.

**Evaluation items**	**Grade I**	**Grade II**	**Grade III**
Vital signs	Unstable	Relatively stable with life support	Stable without life support
Glasgow Coma Scale (GCS) score for consciousness state	Deep coma (GCS score < 9)	Moderate coma (GCS score of 9–12)	No coma or light coma (GCS score > 12)
Respiratory support	Artificial airway with high respiratory support conditions (PEEP ≥ 8 cm H_2_O and FiO_2_ ≥ 60%)	Artificial airway with poor respiratory support conditions (PEEP < 8 cm H_2_O and FiO_2_ < 60%)	Autonomous expectoration without artificial airway support
Circular support	Pumping of ≥ two vasoactive drugs	Pumping of ≥ one vasoactive drugs	No vasoactive drugs required
Primary clinical symptoms	Acute myocardial infarction, severe arrhythmia, severe dyspnea, recurrent convulsions, fatal trauma, and arterial dissection	ECG-suspected myocardial infarction, non-COPD patients with pulse oxygen of < 90%, surgical acute abdomen, severe headache, and severe persistent hyperthermia	Chronic diseases
Medical and nursing staffing	Two physicians and two nurses	One physician and two nurses	One physician and one nurse
Apparatus and drugs	Apparatus: two bottles of oxygen, transport monitor, transport ventilator, oropharyngeal airway, micropump, automated external defibrillator (AED), portable sputum aspirator, intubation supplies, and puncture supplies	Apparatus: one bottle of oxygen, transport monitor, simple ventilator, oropharyngeal ventilator, micropump, AED, and puncture supplies	Apparatus: one bottle of oxygen, handheld pulse oximeter, simple respirator, and puncture supplies
	Drugs: epinephrine, dopamine, amiodarone, midazolam, lidocaine, and atropine	Drugs: epinephrine, midazolam, and saline	Drug: saline

### Observational indicators

In both groups, the average transport time was calculated from the time the patients were transferred from the emergency resuscitation room by the medical staff till they returned to the hospital bed (or arrived at the relevant inpatient unit) after the examination or treatment was completed. The transport time only included the time spent during the transport process, excluding the examination, treatment, and patient handover processes. In addition, we performed intergroup comparisons of the incidence of AEs related to IHT, including the AEs related to the disease (sudden change of consciousness, increase or decrease in heart rate or blood pressure by > 20%, decrease of peripheral oxygen saturation by > 20 or < 90%, sudden cardiac arrest, and massive hemorrhage), personnel (tube dislodgement, displacement, and communication between the teams), and apparatus and drugs (apparatus malfunction [oxygen/respirator failure, black screen, inadequate battery level, non-inflatable cuff, and other technical problems] and inadequate preparation of drugs). Finally, the total length of stay and 30-day intrahospital mortality were compared between the two groups.

### Statistical analysis

IBM SPSS version 25.0 was used for data analyses. Measurements conforming to normal distribution were expressed as mean ± standard deviation (*x* ± SD). An independent samples *t*-test was used to perform intergroup comparisons of the means. Enumeration data were expressed as percentages. In addition, two rates or constituent ratios were compared using the chi-square test, and ranked data were compared using the rank-sum test for multiple ratios. *P* < 0.05 indicated a significant difference.

## Results

### Demographic characteristics

The critically ill patients transported *via* conventional methods (control group) were collected from June 2017 to December 2017 (*n* = 420; mean age, 66.23 ± 19.64 [range, 18–92] years). The observation group included critically ill patients who were transported *via* graded transport from January 2018 to June 2018 (*n* = 380; mean age, 65.45 ± 20.37 [range, 18–94] years). The control group comprised 92, 71, 63, 46, 65, and 83 patients with neurological diseases, cardiovascular diseases, respiratory diseases, digestive system diseases, trauma, and other diseases, respectively. There were 79, 68, 60, 54, 63, and 56 patients with neurological diseases, cardiovascular diseases, respiratory diseases, digestive system diseases, trauma, and other diseases in the observation group, respectively. In addition, no significant intergroup difference was observed in the general information (*P* > 0.05) (Table 3).

**Table 3:** Baseline characteristics of patients.

	**Control group** **(*n* = 420)**	**Observation group** **(*n* = 380)**	***P*-value**
Gender			0.83
Male	244 (58.1%)	224 (58.9%)	
Female	176 (41.9%)	156 (41.1%)	
Age	66.23 ± 19.64	65.45 ± 20.37	0.56
Diseases			0.37
Neurological diseases	92 (21.9%)	79 (20.8%)	
Cardiovascular diseases	71 (16.9%)	68 (17.9%)	
Respiratory diseases	63 (15.0%)	60 (15.8%)	
Digestive system diseases	46 (11.0%)	54 (14.2%)	
Trauma	65 (15.5%)	63 (16.6%)	
Other diseases	83 (19.8%)	56 (14.7%)	
Mortality	74 (17.6%)	63 (16.6%)	0.70

### IHT time of critically ill patients

The IHT time of patients in the observation group from the emergency department to CT/MRI examination, interventional room, surgery room, ICU ward, and general wards was significantly lesser than that of patients in the control group (*P* < 0.05) (Table 4).

**Table 4:** Incidence of adverse events (AEs) in the observation and control groups.

**Groups**	**Disease-related AEs**	**Personnel-related AEs**	**Apparatus-related AEs**	**Drug-related AEs**
Observation group (*n* = 380)	18 (4.73%)	10 (2.63%)	8 (2.11%)	7 (1.84%)
Control group (*n* = 420)	37 (8.81%)	24 (5.71%)	21 (5%)	18 (4.29%)
*x* ^2^	5.1685	4.6589	4.7851	3.9350
*P*-value	0.0230	0.0309	0.0278	0.0473

### Incidence of AEs during the IHT of critically ill patients

One hundred patients in the control group developed AEs during transport (AE incidence rate, 23.81%). In addition, 43 patients in the observation group developed AEs during transport (AE incidence rate, 11.32%). Therefore, there was a significant intergroup difference in the AE incidence (*P* < 0.05). In the control group, the incidence rates of disease-, personnel-, apparatus-, and drug-related AEs were 8.81, 5.71, 5, and 4.29%, respectively, whereas the incidence rates for patients in the observation group were 4.73, 2.63, 2.11, and 1.84%, respectively; both groups showed significant intergroup differences (*P* < 0.05) (Table 5).

**Table 5:** Intrahospital transport time of patients in the observation and control groups.

**Groups**	**CT/MRI examination (*n*)/time (min)**	**Intervention room (*n*)/time (min)**	**Operating room (*n*)/time (min)**	**ICU (*n*)/time (min)**	**General ward (*n*)/time (min)**
Observation group	312/15.64 ± 4.87	42/12.45 ± 2.98	42/14.67 ± 3.54	153/14.89 ± 4.22	227/15.81 ± 5.33
Control group	383/17.83 ± 5.27	49/14.14 ± 3.21	47/16.13 ± 2.82	168/15.96 ± 3.81	252/17.40 ± 5.15
*T*-value	5.6194	2.5856	2.1652	2.3990	3.3195
*P*-value	2.17E-08	0.0113	0.0331	0.0170	0.0010

### Length of stay and 30-day intrahospital mortality rate

The total lengths of stay of patients in the observation and control groups were 19.63 ± 13.95 and 19.18 ± 13.58 days, respectively. Furthermore, the 30-day intrahospital mortality rates were 16.58 and 17.62%, respectively, in the two groups (Table 6). There were no significant intergroup differences in terms of the length of stay and 30-day mortality (*P* > 0.05).

**Table 6:** Length of stay and 30-day intrahospital mortality in the observation and control groups.

**Groups**	**Total length of stay (day)**	**30-day intrahospital mortality**
Observation group (*n* = 380)	19.63 ± 13.95	16.58% (63)
Control group (*n* = 420)	19.18 ± 13.58	17.62% (74)
*t*/*x*^2^	0.4547	0.1521
*P*-value	0.6494	0.6965

## Discussion

IHT is a crucial and unavoidable step for critically ill patients to receive further diagnosis and treatment. However, such patients are susceptible to significant changes during IHT owing to reasons related to disease progression, apparatus used, medications, and transport personnel, which may cause serious complications and could even result in mortality (4). Findings of a recent meta-analysis indicated that critically ill patients may develop AEs during IHT, with a 26.2% incidence rate of mild-to-moderate AEs but a relatively low incidence rate of severe AEs (5). This meta-analysis facilitated a risk-benefit analysis of the diagnostic or therapeutic procedures for critically ill patients requiring IHT. In addition, the incidence rate of AEs was reportedly as high as 79.8% in a study on 441 patients admitted to the ICU undergoing IHT (6). Effective care during IHT is a challenge (7, 8). Owing to the high demand for emergency transport and the exposure to the patient's family during the transport process, medical disputes frequently occur, which places a considerable burden on transport physicians and nurses (9). Consequently, improving the safety of IHT is an area of great interest in research. Overall, meticulous transport planning and procedures, well-performing transport equipment, and well-trained professional teams are necessary to ensure the safety of critically ill patients during transport and reduce the incidence rate of AEs, thereby facilitating the treatment of patients (10–13).

The results of the present study suggested that the IHT time of patients in the observation group from the emergency department to CT/MRI examination, interventional room, surgery room, ICU ward, and the general ward was significantly shorter than that of patients in the control group. This phenomenon could be attributable to the following factors: First, the dedicated transport elevator and staff reduced the waiting time for the elevator; second, transport risk grading allowed medical staff to prepare supplies rapidly and reduce omissions; third, rational manpower allocation and collaboration of medical and nursing teams improved the transport efficiency (14); fourth, communication with medical and technical departments before transport reduced unnecessary waiting time; fifth, changes in the perceptions of medical and technical staff and pre-transport examination of critically ill patients by experienced technicians shortened the transport time. These factors reduced the stress and anxiety of patients and their families and saved the time of medical staff. Furthermore, the reduced waiting time alleviated the lack of oxygen storage, which reduced AEs caused by the low battery level of the monitor and ventilator.

In the present study, the incidence rates of AEs during ITH showed significant intergroup differences, i.e., 23.81 and 11.32% for the control and the observation groups, respectively. In addition, the incidence rate of AEs related to personnel, apparatus, and environment (or hidden problems) was significantly lower in the observation group than in the control group. This phenomenon may be attributed to the following factors. First, after defining and classifying AEs, we changed the perceptions of medical and technical personnel. After realizing the importance of the safety of IHT in the treatment of critically ill patients, the medical and nursing staff carefully evaluated the patients before transport, provided considerable attention to the associated details, and strengthened the assessment of all aspects of the process, which reduced the incidence rate of disease-related AEs. In addition, the selection and use of a graded transport list greatly reduced the lack of pre-transport materials. Furthermore, after theoretical training, operational training, objective structured examinations, and monthly quality control improvements, the medical personnel were calm, prepared, and skilled in the face of emergencies and could appropriately and promptly handle changes related to specific conditions, thereby reducing the incidence rate of personnel-related AEs. Finally, the provision of dedicated transport elevators and operating personnel reduced the unnecessary waiting time, transport time, and power consumption by hypoxia monitors and ventilators, thereby reducing the incidence rate of the environment- and apparatus-related AEs (11).

Studies on the relationship between IHT and the length of stay and mortality in critically ill patients have reported inconsistent findings. Several studies have demonstrated the correlation of the number of IHTs with delirium, length of stay, and mortality (15–17). Meanwhile, Erika et al. (18) observed no significant impact of IHT-related AEs on patient hospitalization. Despite the absence of any significant intergroup differences in terms of the impact of IHT-related AEs on the length of stay and mortality, further measures should be implemented to reduce these adverse factors.

A graded transport model can significantly improve transport efficiency, shorten transport time, and reduce the incidence rate of AEs during the transport process. This mode is a successful innovation in emergency medicine as it can reasonably optimize the allocation of emergency medical resources and improve the safety and efficiency of IHT. Unlike previously used transport modes, the graded transport mode contributes to the establishment of a safe and efficient IHT system for emergency patients. Different transport modes can be applied to corresponding levels of transport to achieve rationalization and safety of medical resource allocation. In addition, effective assessment and grading of the risk of patient transport and prompt preparation according to the actual situation can aid the reasonable allocation of medical resources and further improve the safety of transport, thereby enabling better treatment of critically ill patients.

## Limitations

This study has some limitations. First, the sample size of this study was relatively small. In addition, the retrospective nature of this study may have led to bias in patient selection. Moreover, we could not conduct a survey on the satisfaction of patients and the receiving departments owing to the specificity of the emergency setting. Finally, patient hospitalization costs could not be analyzed in comparative studies. Consequently, further studies are warranted to validate our findings.

## Conclusion

In summary, the graded transport mode has a unique value in clinical settings. It can effectively improve the safety of transport and reduce the incidence of AEs. As a result, vital signs of patients at all risk grades can be effectively maintained during the transport process. Although no significant intergroup differences were observed in terms of the length of stay and 30-day mortality, the graded transport mode can shorten the IHT time of critically ill patients and reduce the AE incidence rates.

## Data availability statement

The original contributions presented in the study are included in the article/supplementary material, further inquiries can be directed to the corresponding author.

## Ethics statement

The studies involving human participants were reviewed and approved by the study was approved by the Ethics Committee of Kunshan Hospital Jiangsu University (Kunshan, China). The patients/participants provided their written informed consent to participate in this study.

## Author contributions

JM and LL conceived, designed, and coordinated the study. LL, JM, and ZG drafted this manuscript. XX, HY, CZ, SL, and JM were responsible for collecting and measuring the imaging data. LL, CZ, XX, and SL were responsible for collecting clinical data. JM and ZG were responsible for data analysis. All authors read, approved, and contributed to the final manuscript.

## References

[1] Bergman LM Pettersson ME Chaboyer WP Carlström ED Ringdal ML. Safety hazards during intrahospital transport: a prospective observational study. Crit Care Med. (2017) 45:e1043–9. 10.1097/CCM.0000000000002653 28787292

[2] Lu KK Zhang MM Zhu YL Ye C Li M. Improving the quality of emergency intrahospital transport for critically ill patients by using Toyota production system methods. J Multidiscip Healthc. (2022) 15:1111–20. 10.2147/JMDH.S360261 35607363 PMC9123905

[3] Brunsveld-Reinders AH Arbous MS Kuiper SG de Jonge E. A comprehensive method to develop a checklist to increase safety of intra-hospital transport of critically ill patients. Crit Care. (2015) 19:214. 10.1186/s13054-015-0938-1 25947327 PMC4438434

[4] Hajjej Z Gharsallah H Boussaidi I Daiki M Labbene I Ferjani M. Risk of mishaps during intrahospital transport of critically ill patients. Tunis Med. (2015) 93:708–13. 27126429

[5] Murata M Nakagawa N Kawasaki T Yasuo S Yoshida T Ando K. Adverse events during intrahospital transport of critically ill patients: a systematic review and meta-analysis. Am J Emerg Med. (2022) 52:13–9. 10.1016/j.ajem.2021.11.021 34861515

[6] Jia L Wang H Gao Y Liu H Yu K. High incidence of adverse events during intra-hospital transport of critically ill patients and new related risk factors: a prospective, multicenter study in China. Crit Care. (2016) 20:12. 10.1186/s13054-016-1183-y 26781179 PMC4717618

[7] Kue R Brown P Ness C Scheulen J. Adverse clinical events during intrahospital transport by a specialized team: a preliminary report. Am J Crit Care. (2011) 20:153–62. 10.4037/ajcc2011478 21362719 PMC3715047

[8] Venn AM Sotomayor CA Godambe SA Vazifedan T Jennings AD Qureshi FA. Implementation of an intrahospital transport checklist for emergency department admissions to intensive care. Pediatr Qual Saf. (2021) 6:e426. 10.1097/pq9.0000000000000426 34235354 PMC8225371

[9] Salt O Akpinar M Sayhan MB Örs FB Durukan P Baykan N. Intrahospital critical patient transport from the emergency department. Arch Med Sci. (2018) 16:337–44. 10.5114/aoms.2018.79598 32190144 PMC7069436

[10] Picetti E Antonini MV Lucchetti MC Pucciarelli S Valente A Rossi I. Intra-hospital transport of brain-injured patients: a prospective, observational study. Neurocrit Care. (2013) 18:298–304. 10.1007/s12028-012-9802-1 23208448

[11] Alizadeh Sharafi R Ghahramanian A Sheikhalipour Z Ghafourifard M Ghasempour M. Improving the safety and quality of the intra-hospital transport of critically ill patients. Nurs Crit Care. (2021) 26:244–52. 10.1111/nicc.12527 32671965

[12] Veiga VC Postalli NF Alvarisa TK Travassos PP Vale RT Oliveira CZ. Adverse events during intrahospital transport of critically ill patients in a large hospital. Eventos adversos durante transporte intra-hospitalar de pacientes críticos em hospital de grande porte. Rev Bras Ter Intensiva. (2019) 31:15–20. 10.5935/0103-507X.20190003 30843950 PMC6443312

[13] Knight PH Maheshwari N Hussain J Scholl M Hughes M Papadimos TJ. Complications during intrahospital transport of critically ill patients: focus on risk identification and prevention. Int J Crit Illn Inj Sci. (2015) 5:256–64. 10.4103/2229-5151.170840 26807395 PMC4705572

[14] Latzke M Schiffinger M Zellhofer D Steyrer J. Soft factors, smooth transport? The role of safety climate and team processes in reducing adverse events during intrahospital transport in intensive care. Health Care Manag Rev. (2020) 45:32–40. 10.1097/HMR.0000000000000188 29176495

[15] Goldberg A Straus SE Hamid JS Wong CL. Room transfers and the risk of delirium incidence amongst hospitalized elderly medical patients: a case-control study. BMC Geriatr. (2015) 15:69. 10.1186/s12877-015-0070-8 26108254 PMC4478641

[16] Perimal-Lewis L Bradley C Hakendorf PH Whitehead C Heuzenroeder L Crotty M. The relationship between in-hospital location and outcomes of care in patients diagnosed with dementia and/or delirium diagnoses: analysis of patient journey. BMC Geriatr. (2016) 16:190. 10.1186/s12877-016-0372-5 27881092 PMC5122028

[17] Schwebel C Clec'h C Magne S Minet C Garrouste-Orgeas M Bonadona A. Safety of intrahospital transport in ventilated critically ill patients: a multicenter cohort study. Crit Care Med. (2013) 41:1919–28. 10.1097/CCM.0b013e31828a3bbd 23863225

[18] Parmentier-Decrucq E Poissy J Favory R Nseir S Onimus T Guerry MJ. Adverse events during intrahospital transport of critically ill patients: incidence and risk factors. Ann Intens Care. (2013) 3:10. 10.1186/2110-5820-3-10 23587445 PMC3639083

